# Editorial: Capping agents encapsulated nanoparticles in plant biotechnology

**DOI:** 10.3389/fpls.2023.1158624

**Published:** 2023-02-16

**Authors:** Rabia Javed, Muhammad Zia, Mumtaz Cheema

**Affiliations:** ^1^ School of Science and the Environment, Memorial University of Newfoundland and Labrador, Corner Brook, NL, Canada; ^2^ Department of Biotechnology, Quaid-i-Azam University, Islamabad, Pakistan

**Keywords:** capping agents, plant nanobiotechnology, nanoparticles, plant extracts, bioreduction

Nanotechnology has been emerged as a promising area of science and its applications has enormous impact on different industries such as agriculture, food, medicine, pharmacy, cosmetics, dentistry, environment, and consequently life of all living organisms. It is the huge versatility of nanomaterials that enables them to be employed in various scientific disciplines. The nanoparticles are occasionally capped with capping agents in order to make them more stable. The capped nanoparticles can be synthesized by chemical and biological methods. The chemical methods require addition of synthetic capping agents while the biological procedures have natural capping agents found in the extracts that perform dual function of reducing and capping agents. For instance, biomolecules like antioxidants (polyphenols, terpenoids, carotenoids), fatty acids, lipids, amino acids, proteins, carbohydrates, and vitamins found in plant extracts are capable of both reduction and capping of metal ions. The major constraint for implementation of nanotechnology in any domain of biotechnology is the potential toxicity and risk hazards associated with it. So far, different techniques have been adopted to fabricate capped nanoparticles to enhance their applications and overcome the rising challenges of bioaccumulation and toxicity of nanomaterials. The stabilizing agents are intended to do wonders due to their extraordinary shelf life and functional surfaces enhancing their applicability in different agro-biological systems.

There is an exponentially increasing need to fabricate novel nanoparticles to be applied as vehicles for efficient gene and nutrients’ delivery and for performing key roles in plant biotechnology for biotic/abiotic stress tolerance and to fix various pathological disorders. The previous literature provides very little information on the exploitation of potential approaches of capped nanomaterials and further research is required in this innovative domain. Therefore, the remarkable abilities of surface modification of nanoparticles should be explored. The goal of exploring the effective employment of surface functionalized nanoparticles in plant biotechnology has been achieved in research articles published in this Research Topic.

Ahmad et al. investigated the impact of biosynthesized silver (Ag) nanoparticles on the control of fusarium wilt in *Solanum lycopersicum* (tomato). This study developed and evaluated the nanopesticides having significant antifungal properties. The aqueous extract of *Polygonatum geminiflorum* having biological capping agents was utilized for producing 27 nm sized stable Ag nanoparticles that were characterized using transmission electron microscopy (TEM), x-ray diffraction (XRD), fourier transform infrared spectroscopy (FTIR), UV-visible spectrophotometry, and energy dispersive x-ray (EDX) techniques. The phytochemical and antioxidant assays of *P. geminiflorum* extract revealed total phenolic and flavonoid content as well as the 2,2-diphenylpicrylhydrazyl (DPPH) radical scavenging activity by spectroscopic technique. The high performance liquid chromatography (HPLC) was performed to find secondary metabolites profile of *P. geminiflorum* extract that revealed the presence of 15 bioactive phenolic compounds. *In vitro* and in planta studies of Ag nanoparticles were performed *via* well-diffusion/disc-diffusion assays and foliar spray method under controlled conditions against *Fusarium oxysporum*. The results revealed 100% inhibition of Fusarium wilt of tomato, hence protecting crop health using bio-control agents.

Rafiq et al. determined the influence of biofabricated zinc oxide (ZnO) nanoparticles on *Vigna radiata* (mung bean) plant having Cercospora leaf spot disease. The nanopesticides against fungal pathogen were developed and evaluated by *in vitro* and in planta studies. The fungal strain isolated from infected leaf was identified to be *Cercospora canscens*. The seed extract of *Trachyspermum ammi* was used for biological synthesis of ZnO nanoparticles that were characterized by XRD, FTIR, SEM, and UV-visible spectrophotometry. *In vitro* experiment was conducted using disc-diffusion technique to study antifungal potential of ZnO nanoparticles. In planta experiment resulted in not only the control of leaf spot disease but also substantially improved the plant growth parameters as well as carotenoid and chlorophyll content. In this way, plant disease management was achieved.

Beig et al. explored the effect of ZnO nanoparticles coated with molasses and bound with urea fertilizer on the growth and development of *Triticum aestivum* (Wheat). The slow-release coated ZnO nanofertilizers were developed and tested for their improvement in N and Zn use efficiency. The ZnO nanoparticles were synthesized by chemical method and coated with molasses, then characterized by SEM, XRD, and FTIR. In planta experiments evaluated urea N release rate, water leaching, and crushing strength. The boost in growth and yield of wheat was observed under different treatments that occurred by the reduced release of N and Zn. Sustainable crop yield was achieved by the effective use of coated nanofertilizers at the lower dosage which acted as potential cost saver by minimizing the chemcials’ input into the environment.

Salih et al. studied the influence of biogenic Ag nanoparticles on the improvement of bioactive compounds of medicinal plant *Juniperus procera*. Ag nanoparticles were biosynthesized from the aqueous leaf extract of *Phoenix dactylifera* and characterized using UV-visible spectroscopy, FTIR, SEM, and dynamic light scattering (DLS). Phytochemicals of *P. dactylifera* were screened by gas chromatography mass spectrometry (GC-MS). The callus of *J. procera* grown *in vitro* under different Ag nanoparticles treatments revealed their significant impact on biomass accumulation and enzymatic and non-enzymatic antioxidants. Moreover, bioactive compounds quantified using HPLC were substantially increased. Hence, improvement in growth and phytochemical profile of *J. procera* was achieved by nanoelicitation.

All these studies employed biological approach for nanoparticles’ synthesis except a single study that involved nanofabrication by chemical route. This is due to the emerging trend towards biogenic synthesis of nanoparticles as it is easy, economical, and eco-friendly that produces nanoparticles having longer shelf life, more biocompatibility, and enhanced biological effect. In short, some of the research gaps have been addressed by the research articles published in this Research Topic while few still need attention.

## Author contributions

All authors listed have made a substantial, direct, and intellectual contribution to the work and approved it for publication.

